# *OPA1* gene therapy prevents retinal ganglion cell loss in a Dominant Optic Atrophy mouse model

**DOI:** 10.1038/s41598-018-20838-8

**Published:** 2018-02-06

**Authors:** Emmanuelle Sarzi, Marie Seveno, Camille Piro-Mégy, Lucie Elzière, Mélanie Quilès, Marie Péquignot, Agnès Müller, Christian P. Hamel, Guy Lenaers, Cécile Delettre

**Affiliations:** 1 UMR INSERM U1051/Université Montpellier - Institut des Neurosciences de Montpellier, 34091 Montpellier, France; 2 Université de Montpellier - Faculté de Pharmacie, 34093 Montpellier, France; 3 Affections sensorielles génétiques, Hôpital Gui de Chauliac, Montpellier, France; 4 PREMMI, UMR CNRS 6015, INSERM U1083, Université d’Angers, Angers, France

## Abstract

Dominant optic atrophy (DOA) is a rare progressive and irreversible blinding disease which is one of the most frequent forms of hereditary optic neuropathy. DOA is mainly caused by dominant mutation in the *OPA1* gene encoding a large mitochondrial GTPase with crucial roles in membrane dynamics and cell survival. Hereditary optic neuropathies are commonly characterized by the degeneration of retinal ganglion cells, leading to the optic nerve atrophy and the progressive loss of visual acuity. Up to now, despite increasing advances in the understanding of the pathological mechanisms, DOA remains intractable. Here, we tested the efficiency of gene therapy on a genetically-modified mouse model reproducing DOA vision loss. We performed intravitreal injections of an Adeno-Associated Virus carrying the human *OPA1* cDNA under the control of the cytomegalovirus promotor. Our results provide the first evidence that gene therapy is efficient on a mouse model of DOA as the wild-type *OPA1* expression is able to alleviate the *OPA1*-induced retinal ganglion cell degeneration, the hallmark of the disease. These results displayed encouraging effects of gene therapy for Dominant Optic Atrophy, fostering future investigations aiming at clinical trials in patients.

## Introduction

Hereditary optic neuropathies define a group of blinding diseases characterized by a progressive degeneration of the retinal ganglion cells (RGC)^1^. They are mainly due to modifications in genes encoding ubiquitous mitochondrial proteins and their pathological mechanisms responsible for the specific RGC degeneration remain poorly understood^2^. Dominant optic atrophy (DOA) is one of the most frequent forms of these diseases, and is mainly related to dominant *OPA1* mutations^3^. Importantly, in the majority of DOA cases, haplo-insufficiency of the OPA1 protein represents the princeps mechanism responsible for the disease^4^ supporting that gene supplementation should be beneficial. Up to now, while gene therapy trials are under process in patients with the maternally-inherited Leber’s optic neuropathy, the other major hereditary optic neuropathy, no gene therapy approach has been reported for the treatment of DOA^5^. In the last years, we generated and characterized a DOA mouse model carrying the most frequent *OPA1* mutation that faithfully reproduces the syndromic form of DOA^6,7^. Based on this model, we have appraised the efficiency of gene therapy involving the transduction of adeno-associated virus carrying the human wild-type *OPA1* cDNA under the control of the Cytomegalovirus (CMV) promotor. Our results show that *CMV*-induced *OPA1* expression prevents RGC loss in *Opa1*^+/−^ treated mice.

## Results

**Figure 1:**
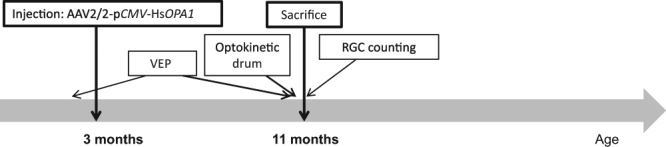
*Gene therapy strategy*. p*CMV*: *CMV* promotor; Hs*OPA1*:Homo sapiens *OPA1*; VEP: Visual Evoked Potentials; RGC: retinal ganglion cells. This strategy included 4 female *Opa1*^+/+^ controls, 4 untreated and 8 treated female *Opa1*^+/−^ mice.

We designed a strategy based on the injection of an AAV2 serotype 2 known to transduce specifically retinal ganglion cells^8^ (Fig. 1). In this vector, the human isoform #1 *OPA1* full length cDNA was incorporated and its expression was driven by the CMV promotor. To control the level of the human *OPA1* (Hs*OPA1*) gene expression, we transfected murine NIH3T3 cells with the pGG2 vector carrying the p*CMV*-Hs*OPA1* construction (Supplementary Figure S1). While the endogenous murine *Opa1* gene was found expressed in untransfected, empty-pGG2 and pGG2-p*CMV*-Hs*OPA1* transfected cells, we found that Hs*OPA1* transcripts were quantified only in pGG2-p*CMV*-Hs*OPA1* transfected cells (Figure S1). Hs*OPA1* expression was found larger than the endogenous *Opa1* gene (Figure S1A), which has been confirmed with the OPA1 protein expression monitored by western blot (Figure S1B). Thus, these *in vitro* experiments validated that the *CMV* promotor is able to efficiently enhance the Hs*OPA1* gene production.

**Figure 2:**
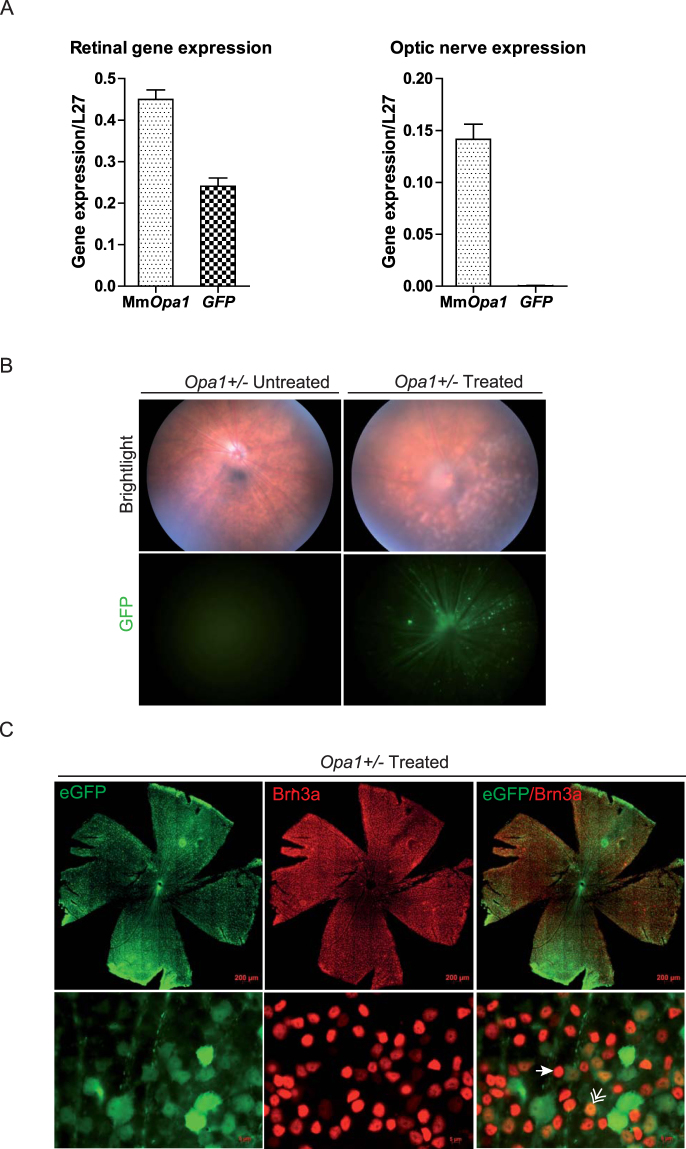
*Assessment of the vector transduction efficiency*. (**A**) Quantification of murine endogenous *Opa1* transcripts as well as *GFP* transcripts. Mm*Opa1*: Mus musculus *Opa1* (**B**) *In vivo* funduscopy was performed on untreated and treated *Opa1*^+/−^ mice in brightfield (upper panel) and using a specific green filter to observe GFP fluorescence (lower panel). (**C**) Post-mortem immunohistochemistry was done on whole mount retinas using a RGC specific Brn3a labelling (Upper panel). Zoom-in (lower panel) was done to observe Brn3a/GFP double-positive RGCs (double arrow), Brn3a positive (arrow) and GFP negative RGCs.

Then, a preliminary *in vivo* analysis using the AAV2/2-*CMV-GFP* construction was used to follow the transduction efficiency and long-lasting expression of this vector after injection (Fig. 2). We first quantified in control mice the *GFP* and the endogenous *Opa1* gene expressions. Thus, using real-time quantitative PCR we monitored these gene expressions in retina as well as in optic nerve as negative control, 2 months after intravitreal injections (Fig. 2A). We found the expression of the murine *Opa1* gene in both retina and optic nerve. By contrast, *GFP* transcripts were found expressed in retina but not in optic nerve. Then, we observed by *in vivo* funduscopy the presence of GFP positive retinal areas (Fig. 2B) and found that GFP positive cells are homogeneously distributed on whole mounted retinas. Co-immunolabelling with the Brn3a RGC specific marker showed that 42.9% ±2.03 of Brn3a positive cells were also GFP positive. Moreover, we found that 83% of the total number of GFP positive cells were Brn3a positive (Fig. 2C, Supplementary Figure S2).

**Figure 3:**
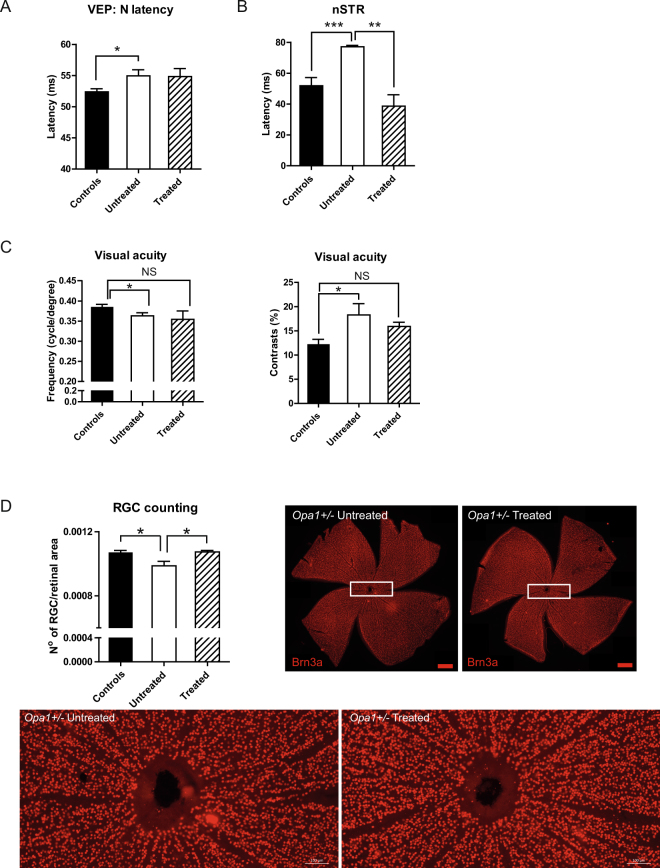
*Efficiency of the AAV2/2 injection on visual function and retinal ganglion cell number*. (**A** and **B**) *In vivo* electrophysiological recordings. (**A**) visual evoked potential (VEP) N-wave latencies, (**B**) negative scotopic threshold response (nSTR). (**C**) Assessment of visual acuity using an optokinetic drum. Measurements of frequency thresholds (left) and contrast perception (right). NS: not significant. (**D**) post-fixed RGC counting using a specific Brn3a immuno-labelling in whole mount retinas. White rectangles: enlargement of whole-mount retinas. Brn3a positive dots were quantified using Imaris and MetaMorph® softwares. Data were expressed as the mean ± SEM. *P < 0.05, **P < 0.01, ***P < 0.001 using one-way ANOVA.

We further performed intravitreal injections of the AAV2/2-p*CMV-HsOPA1*, carrying the human variant #1 *OPA1* cDNA full length transcript on 3-month-old *Opa1* mice, before the onset of progressive degenerative processes in retina^6,7^. Eight months following injection, we assessed the quality of the visual pathways from retinal ganglion cells to cortical areas by recording visual evoked potentials. As previously reported, we found a significant increase of the N-wave latency in untreated mice compared to controls (Fig. 3A). In consequence to treatment, *Opa1*^+/−^ mice harbored similar increase of the N-wave latency as found in untreated mice. In addition, we analysed the scotopic threshold responses (STRs) recorded using flash-electroretinograms, that assess the activity of inner retina neurons^9^. We found a significant increase in the negative STR (nSTR) latencies of heterozygous untreated mice compared to controls (Fig. 3B). Interestingly, the nSTR latency values obtained after treatment were drastically decrease reaching those recorded in control animals (Fig. 3B). In parallel, we examined *in vivo* the outcome of the AAV2/2 injection on mice visual acuity. In agreement with our previous data^7^, *Opa1*^+/−^ untreated mice developed a loss of visual acuity, revealed by the decrease of the frequency thresholds and the increase of the contrast perception. Interestingly, in treated *Opa1* mice we found an increase of the frequency threshold comparable to that of controls (Fig. 3C). In the same way, contrast perception threshold was slightly decreased in treated *Opa1*^+/−^ mice compared to untreated animals reaching level observed in control mice. Following the *in vivo* exploration, mice were sacrificed and we performed the RGC counting on whole mount retinas using the specific Brn3a RGC labelling. As previously described at 11 months, *Opa1*^+/−^ untreated mice harbored a loss of RGCs compared to controls. In consequence to treatment, the number of RGC in *Opa1*^+/−^ mice was significantly increased compared to untreated mice and reached control values (Fig. 3D).

## Discussion

Our results showed the efficiency of the AAV2 construction to transduce and maintain *OPA1* expression in the mouse retinal ganglion cells. Moreover, injection of the construction carrying the human variant #1 *OPA1* cDNA, which gives rise to both long and short OPA1 isoforms, driven by the *CMV* promotor prevents RGC progressive degeneration, which is the hallmark of the disease.

Beyond the prevention of retinal ganglion cell degeneration, we showed the benefit of *OPA1* gene therapy on the electrophysiological measurements specific of retinal ganglion cell activity. Indeed, although visual evoked potentials (VEPs) recorded did not show any difference 9 months after injection, the analysis of STRs revealed a clear benefit of the treatment on retinal ganglion cell activity. VEPs are basically used to assess whether RGC axons are capable to carrying retinal signals along the geniculo-cortical pathway. On the other hand, STRs remain a more sensitive protocol to investigate inner retinal circuits and thus retinal ganglion cell activity. All together, these two electrophysiological approaches emphasized a dysfunction of retinal ganglion cells in *Opa1* untreated mice. But interestingly, the effect of gene therapy improves more the retinal ganglion cell intra-retinal functioning rather than their capability to carry the electric signal to the cortical visual areas. These results are supported by those provided by the assessment of visual acuity *in vivo*. Indeed, visual acuity, which is the major parameter to appreciate visual capacities, remains slightly improved in *Opa1*^+/−^ treated mice. Thus, our results show that *OPA1* gene therapy may be sufficient to prevent retinal ganglion cell degeneration but not enough to efficiently maintain the conduction of the electric information to the visual perception zones. Interestingly, a discrepancy between cell loss prevention and electrophysiological function has been already described in the case of *RPE65* gene therapy trials in both animals and patients with the Leber’s congenital amaurosis^10^. We can consider that the number of transfected retinal ganglion cells remains insufficient to trigger appreciable improvement of the electric activity along the optic path. Indeed, the amount of Brn3a positive RGCs without GFP expression suggests the existence of a cell reservoir that could be transduced with higher doses of viral genomes. Indeed, this could also explain why the level of GFP protein found in retina is significantly lower than the endogenous *Opa1* expression. Nevertheless, this level of transduction remained efficient to prevent retinal ganglion cell dysfunction and degeneration.

These first gene therapy results established the hints that *OPA1* expression in retinal ganglion cells can be an effective solution to treat dominant optic atrophy. This provides challenging perspectives to go further on the improvement of trials to efficiently develop pre-clinical trials for this blinding disease that nowadays remains without any treatment.

## Material and Method

### Opa1^delTTAG^ mouse model

The *Opa1* knock-in mice carrying the c.2708_2711delTTAG mutation was described in Sarzi *et al*.^6^. Mice were kept in the animal facility of the Institute for Neurosciences of Montpellier (B 3417236, 11/03 2010). All protocols were carried out on *Opa1*^+/−^ mice and their *Opa1*^+/+^ littermates.

### Intravitreous injections

Mice were anesthetized by intra-peritoneal injections of a mixture of Ketamine (120 mg/kg) and Rompun (10 mg/kg). The viral constructs used in this study were produced at Laboratoire d’Amplification de Vecteurs – CHU de Nantes, based on the provided sequences, and stocked at −80 °C until use. We introduced the human variant #1 *OPA1* cDNA full length under the control of the *CMV* promotor. Two µl of the AAV2/2-p*CMV-HsOPA1* or AAV2/2-p*CMV-GFP* vector were injected intra-vitreally at a titration of 1.6 10^11^ vg/ml or 3.2 10^8^ per injection. The injected eye was covered with ophthalmic gel (Lacryvisc; Alcon®, Novartis; Fort Worth, USA). After installing a circular coverslip, the eye was pierced with a 34-gauge needle (Hamilton MicroSyringe; Reno, USA) for pressure equalization. Then, a Hamilton 5 μl syringe (Microliter^TM^ #65; Hamilton MicroSyringe; Reno, USA) with a blunt 34-gauge needle was used for gene delivery. Vector injection was done on the opposite side of the first hole, and the needle was kept inside several minutes until the eye coloring returned to normal. Finally, the needle was slowly removed. The mouse was kept on a heating plate at 37 °C, with the eye covered by the ophthalmic gel, and was kept in a separate cage until full recovery. Experiments were developed in accordance with the ARVO (Association for Research in Vision and Ophthalmology) Statement for the Use of Animals in Ophthalmic and Visual Research. Injections of the AAV2/2-p*CMV-HsOPA1* or the AAV2/2-p*CMV-GFP* vectors were carried out on *Opa1* mice in this proportion: 4 *Opa1*^+/+^ female controls, 4 untreated and 8 treated *Opa1*^+/−^ female mice. Five control mice were injected with the AAV2/2-p*CMV*-*GFP* construction to quantify GFP expression in retina and optic nerve.

### Quantitative real-time PCR

Quantitative real-time polymerase chain reactions were performed on total RNA extracted from retinas and optic nerves using the RiboPureTM Kit (Ambion). One microgram of RNA product was reverse-transcribed with the VersoTM cDNA Kit (Thermo Fisher Scientific) according to the manufacturer’s instructions. The endogenous murine *Opa1* transcripts as well as the human transduced *OPA1* transcripts were quantified using a specific pair of primers with SYBR_ Green/LightCycler_ technology (Roche). Primers for murine *Opa1* transcripts quantification: F: CAGGAGAAGTAGACTGTGTC, R: TGTGACTTTATTTTGCACGG. Primers for *GFP* transcripts: F: AGTGCTTCAGCCGCTACCC and R: CAGCTCGATGCGGTTCACC. Primers for human *OPA1* transcripts: F: GCAATTGAAAACATGGTGGGT and R: CTGGGTGCTCCTCATTACAT. Primers for the ribosomal protein *L27* gene: F: ACGCAAAGCCGTCATCGTGAAG and R: CTTGGCGATCTTCTTCTTGCC. Gene expressions were reported to the L27 gene expression of each sample.

### Visual electrophysiology

Electrophysiology was performed in a darkroom, in the morning after overnight dark-adaptation, as advised^11^. Visual Evoked Potentials (VEPs) were performed on anesthetized mice, and recorded using three phases of 60 flashes as previously described^12^. Flash duration was 5 milliseconds with a frequency of 1 Hz and intensity of 159 cd.s^−1^.m^−2^. Amplitudes and latencies obtained during each phase were averaged. A cut-off filter was set at 35 Hz. For flash-electroretinogram acquisition mice were exposed to flashes of light lasting 5 ms at 0.1 Hz frequency each, with growing intensities (0.159, 0.3, 0.5, 1.59, 5, 15.9 cd∙s/m^2^); for the scotopic ERG. That included the scotopic threshold responses (STRs) of retinal ganglion cells^9^, as the second of six recordings. Electrophysiological data were analyzed with Visiosystem software (SIEM BioMedicale; Nîmes, France).

### Virtual-reality optokinetic system

Mouse visual acuity was assessed as described by Prusky *et al*.^13^. The apparatus included a virtual optomotor constituted by four computer monitors arranged in a quadrangle arena generating a virtual rotating cylinder with drifting vertical sine wave grating. The OptoMotry (Cerebral Mechanics, Lethbride, Alberta, Canada) was used to control grating parameters and video recordings. Mice were placed on a central platform allowing them to track the grating with reflexive head and neck movements. The increasing of the grating frequency (from 0.042 to 0.442 cyc/deg.) until the optomotor reflex was not detectable anymore, defined the visual acuity. Contrast sensitivity was given at the 0.042 cyc/deg frequency. Mice were tested for both eyes in the morning at the beginning of their daylight cycle. Experiments were done blind to the genotype.

### Funduscopy

*In vivo* funduscopy was performed using a Micron III retinal-imaging microscope (Phoenix Research Laboratories, Pleasanton, CA). Mice were anesthetized by intra-peritoneal injections of a mixture of Ketamine (120 mg/kg) and Rompun (10 mg/kg) and pupils were dilated with 1% tropicamide. The Lacryvisc® corneal ophthalmic gel (Alcon, Fort Worth, TX) was added before starting. Bright field and a specific green filter were used to observe retinal fundus and GFP expression, respectively. Still-frame images and video-rate sequences were acquired with Streampix III image acquisition software (NorPix, Montreal, Canada).

### Retinal ganglion cell counting

Mice were euthanized by cervical dislocation. Eyes were collected and neuroretinas were isolated and fixed in paraformaldehyde 4%. For RGC counting, whole neuroretinas were immunolabelled using a Brn3a primary antibody (1:500, Santa Cruz Biotechnology) along with an Alexa 594 conjugated anti-goat secondary antibody (1:800, Invitrogen, Oregon, USA). Four lateral sections were done on retinas to allow their correct unfolding and mounting between slide and coverslip. Whole-mount retina images were obtained using the right Axioimager Z1 microscope with ApoTome module (Zeiss). Retinal ganglion cell number was calculated by the Imaris software and related to the retina surface calculated by the MetaMorph® Offline software.

### Statistical analysis

Mann-Withney U-test and one-way ANOVA followed by the Student-Newman-Keuls multiple comparison test were used. Statistical significance thresholds were determined at *P* < 0.05 (*), *P* < 0.01 (**) and *P* < 0.001 (***) values.

### Study approval

All protocols carried out on animals were approved by the Animal Care and Use Committee Languedoc-Roussillon and recorded under the reference: CEEA-LR-11058, in agreement with the ARRIVE guidelines. All efforts were made to minimize the number of used animals and their suffering, according to the European directive 2010/63/UE. The authors confirm that they are in compliance with their Institutional Review Boards (IRBs).

## Electronic supplementary material

Supplementary Information
