# Supplementary material for: OPA1 gene therapy prevents retinal ganglion cell loss in a Dominant Optic Atrophy mouse model
Source: Sci Rep. 2018 Feb 6;8:2468. doi: 10.1038/s41598-018-20838-8 (PMC5802757; doi:10.1038/s41598-018-20838-8)
Supplement: Supplementary file 1 — Supplementary Information [file 41598_2018_20838_MOESM1_ESM.pdf]

SUPPLEMENTARY INFORMATION

OPA1 gene therapy prevents retinal ganglion cell loss in a Dominant Optic Atrophy mouse model.

Emmanuelle Sarzi[1], Marie Seveno[1], Camille Piro-Mégy[1], Lucie Elziere[1], Mélanie Quilès[1], Marie Péquignot[1], Agnès Müller[1,2], Christian P Hamel[1,3], Guy Lenaers[1,4*], Cécile Delettre[1*].

[1]UMR INSERM U1051/ Université Montpellier - Institut des Neurosciences de Montpellier, 34091 Montpellier, France.

[2]Université de Montpellier - Faculté de Pharmacie-34093 Montpellier, France

[3]Affections sensorielles génétiques, Hôpital Gui de Chauliac, Montpellier, France

[4]PREMMI, UMR CNRS 6015, INSERM U1083, Université d'Angers, France

* Last authors contributed equally to this work

Corresponding author : Dr Emmanuelle Sarzi, PhD, Institut des Neurosciences de Montpellier, Hôpital St Eloi – 34091 Montpellier – France, e-mail: emmanuelle.sarzi@inserm.fr

SUPPLEMENTARY FIGURES

Figure S1

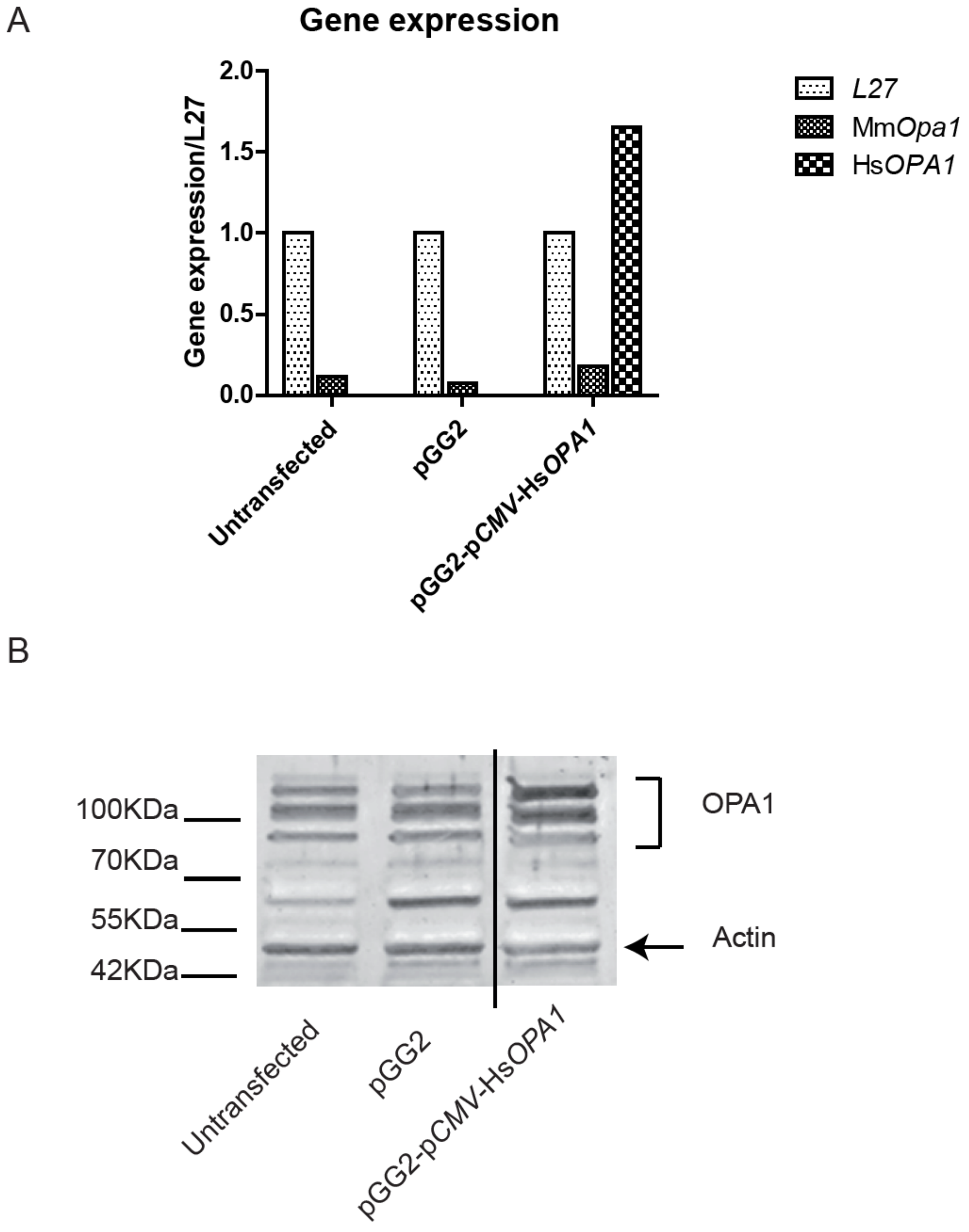

Figure S1: Control of the Hs*OPA1* gene and protein expression in the NIH3T3 mouse strain. (A) real-time quantitative PCR of murine *Opa1* and Hs*OPA*1 transcripts reported to the L27 gene expression (*n*=3). (B) Western blot using a home-made-OPA1 and actin antibodies.

Figure S2

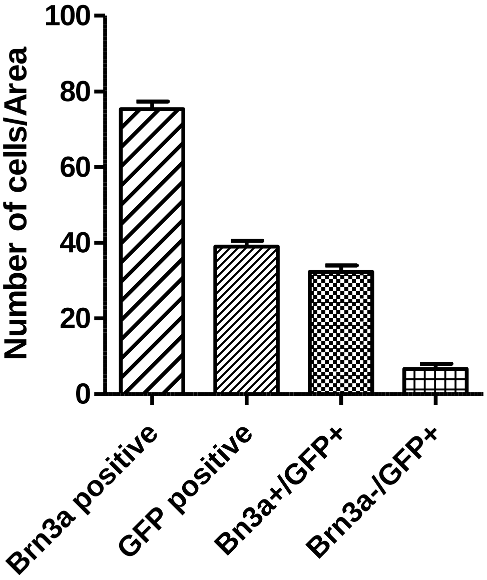

Figure S2: Quantification of GFP and Brn3a staining on whole-mount retina. Data were expressed as the mean ± SEM.

SUPPLEMENTARY MATERIAL AND METHOD

NIH3T3 transfections and control of the HsOPA1 protein expression

NIH3T3 cells were cultured in complete culture medium (Dulbecco's modified Eagle's medium, supplemented with 10% fetal calf serum and 100 units/ml penicillin/streptomycin, Invitrogen) and maintained at 37 °C in a humidified 5% CO2 atmosphere. Cells were seeded at a concentration of a 2×10^5 into 6-well plates. Transient transfections with the pGG2-p*CMV*-Hs*OPA1* construction were performed using Lipofectamine 2 000 in Optimem (Invitrogen) according to the manufacturer's protocol. Empty pGG2 vector was used as control. Twenty-four hours after transfection, cells were harvested and protein were extracted in RIPA buffer (50 mM Tris–HCl, pH8.0, 150 mM sodium chloride, 1% NP-40, 0.1% SDS, and 0.5% sodium

deoxycholate). Proteins (40 μg) were separated by 7.5% SDS-PAGE and transferred onto a nitrocellulose membrane (Bio-Rad, Hertfordshire, UK). Primary antibodies against OPA1 protein (home-made antibody) and Actin (1:5000, Sigma-Aldrich) were visualized using horseradish peroxidase-conjugated secondary antibody.
